# Emerging clinical and research approaches in targeted therapies for high-risk neuroblastoma

**DOI:** 10.3389/fonc.2025.1553511

**Published:** 2025-03-04

**Authors:** Albatool AlKhazal, Samiha Chohan, Destani J. Ross, Jinhwan Kim, Erin G. Brown

**Affiliations:** ^1^ Department of Surgery, School of Medicine, University of California, Davis, Davis, CA, United States; ^2^ Department of Biological Sciences, California State University, Sacramento, Sacramento, CA, United States; ^3^ Department of Biomedical Engineering, University of California, Davis, Davis, CA, United States

**Keywords:** high-risk neuroblastoma, neuroblastoma biology, targeted therapy, pediatric cancer, clinical and preclinical research

## Abstract

Neuroblastoma is a pediatric cancer that originates from neural crest cells and is the most common extracranial solid tumor in children under five years of age. While low-risk neuroblastoma often regresses spontaneously, high-risk neuroblastoma poses a significant clinical challenge. Recent advances in understanding neuroblastoma’s molecular mechanisms have led to the development of targeted therapies that aim to selectively inhibit specific pathways involved in tumor growth and progression, improving patient outcomes while minimizing side effects. This review provides a comprehensive review of neuroblastoma biology and emerging therapeutic strategies. Key topics include (a) immunotherapies and immunotargets, (b) non-coding RNAs (long non-coding RNA, microRNA, and circular RNA), (c) molecular biomarkers and pathways, and (d) limitations and future directions.

## 1 Introduction

Neuroblastoma is the most common extracranial solid tumor in children under the age of five, accounting for approximately 15% of childhood cancer-related deaths (1). It originates from immature neural crest cells within the sympathetic nervous system (2). More specifically, neuroblastoma is thought to arise from sympathoadrenal progenitor cells, which first emerge in the sympathetic ganglia and adrenal glands during development. These progenitor cells co-express markers indicative of both sympathetic neurons and chromaffin cell differentiation (3). During normal development, sympathoadrenal (SA) cells migrate to their designated locations, where extrinsic signals guide their differentiation into either sympathetic neurons or chromaffin cells (4). However, when this differentiation process is disrupted, tumors can form along the sympathetic nervous system, including in the adrenal gland and paraspinal ganglia from the head and neck down to the pelvis (5, 6).

Neuroblastoma is often referred to as a “tumor of extremes” due to its biological heterogeneity, manifested in the presence of two primary cell types: undifferentiated mesenchymal cells and more differentiated adrenergic cells (7). These cell types can switch back and forth (interconvert), resembling cells at different stages of normal neural crest development (7). This cellular diversity likely underlies the disease’s highly variable clinical course, ranging from spontaneous regression in some cases to aggressive, fatal progression in others. This variability has intrigued researchers since its discovery (8, 9). Neuroblastoma’s Neuroblastoma was first identified as a pediatric cancer in the mid-nineteenth century, and its neuronal origin was established by Wright in 1910 (10). Following the emergence of pediatric surgery after World War I, Robert Gross perfected surgical techniques for children and reported on over 200 neuroblastoma cases (11). In collaboration with radiologist Martin Wittenborg, Gross used orthovoltage X-ray therapy to achieve local control of the disease (12). They discovered that older patients with advanced disease had poorer outcomes compared to younger patients, who had more favorable survival rates. By the mid-20^th^ century, chemotherapy, radiotherapy, and surgery became the standard of care, and in 1971, a formal staging system for the neuroblastoma was established (13).

Neuroblastoma patients are categorized into low-, intermediate-, and high-risk groups based on clinical, pathologic, and genetic factors, including tumor stage, age at diagnosis, and molecular markers (14). High-risk neuroblastoma (HRNB) constitutes approximately 50-60% of all neuroblastoma cases and is associated with poor prognostic indicators such as MYCN amplification, metastatic disease, and unfavorable histology. Despite significant advancements in treatment, outcomes for children with HRNB remain poor, with a 5-year overall survival (OS) rate ranging from 51-62% (14). Tumor heterogeneity and development of treatment resistance in HRNB present significant therapeutic challenges (15). However, with advancements in biotechnology and sequencing technologies, precision medicine has emerged as a promising approach for improving outcomes, particularly through targeted therapies aimed at the molecular drivers of the disease.

**Figure 1:**
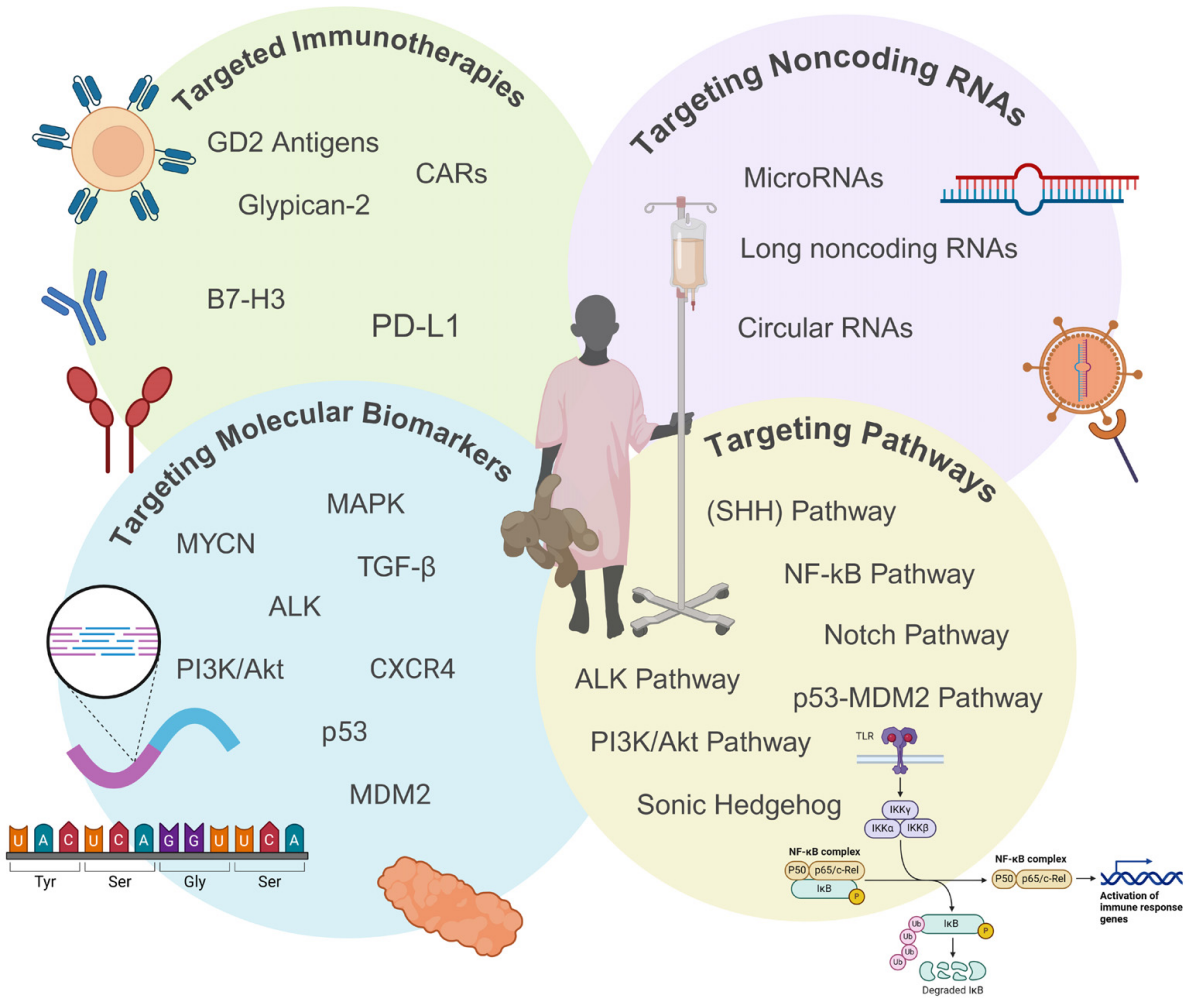
Overview of emerging targeted therapeutic approaches for high-risk neuroblastoma (HRNB). This figure highlights emerging targeted therapies for HRNB, including immunotherapies, noncoding RNAs, biomarkers, and dysregulated pathways. These approaches aim to overcome the limitations and toxicities of traditional treatments by offering more precise and effective therapeutic options to improve outcomes for HRNB patients. *Created with BioRender*.

The current treatment approach for HRNB includes intensive multimodal and systemic regimens such as chemotherapy, immunotherapy, and stem cell transplantation. These systemic therapies damage both cancerous and healthy cells and notoriously result in significant short and long-term adverse effects, negatively impacting the quality of life for patients. This underscores the need for more effective and targeted therapies that address the underlying molecular mechanisms of the disease (16). Targeted therapies may target proteins expressed on cancer cells or address mutations in DNA or protein expression patterns. Mechanisms of action for targeted therapies include blocking chemical signaling pathways or delivering toxins directly to cancer cells, all of which work to suppress tumor cell proliferation. As genome-wide sequencing continues to uncover more molecular biomarkers specific to these heterogeneous tumors, targeted and precision medicine strategies can be further refined, ultimately improving outcomes and reducing the cytotoxicity of current treatments. This review focuses on understanding the biology of HRNB and explores ongoing research and clinical strategies aimed at developing and integrating targeted therapies (**Figure 1**).

## 2 Understanding high-risk neuroblastoma biology and current standard of care

### 2.1 Defining high-risk neuroblastoma

Due to neuroblastoma’s variable clinical spectrum, risk stratification plays a crucial role in determining treatment plans and predicting outcomes. Historical staging systems have been replaced by the International Neuroblastoma Risk Group (INRG) Staging System introduced in 2005, which allows for preoperative risk stratification and incorporates the assessment of image-defined risk factors (IDRFs) to distinguish non-metastatic tumors into stages L1 and L2 (17). L1 tumors represent tumors without evidence of encasement or compression of critical nerves, blood vessels, or organs; while the presence of these imaging features indicate an L2 tumor. Tumors with IDRFs have a higher risk of disease recurrence, more challenging surgical resection and overall poorer outcomes. Children with metastatic disease are classified as stage M; while children under 18 months of age with metastasis limited to the skin, liver, or bone marrow are a unique subset of patients classified as stage MS. In addition to the INRG stage, factors such as the patient’s age at diagnosis, tumor histology and cytogenetic features are used to further refine risk classification. In 2021, the Children’s Oncology Group (COG) added segmental chromosomal aberrations (SCAs) to the risk assessment, as these aberrations are associated with poor outcomes (14).

Revisions to the neuroblastoma risk classification by COG reflect the impact of ongoing research efforts to better understand neuroblastoma tumor biology and advancements in predicting prognosis. However, despite these efforts, HRNB remains a major treatment challenge, with a 5-year event-free survival (EFS) rate of just 51% for HRNB patients, compared to over 85% for those in low- and intermediate-risk groups (14).

### 2.2 Current standards of care for HRNB

#### 2.2.1 Induction phase

The treatment of HRNB typically involves three phases: induction, consolidation, and post-consolidation. The induction phase aims to eliminate visible disease and achieve remission, using five to eight cycles of intensive chemotherapy with drugs such as platinum agents, topoisomerase inhibitors, and alkylating agents. Ongoing COG trials utilize a combination of topotecan, vincristine, doxorubicin, cisplatin, cyclophosphamide, and etoposide chemotherapeutics (18). Autologous stem cell collection occurs during induction, as stem cells are purged of malignant cells before being stored for later use in consolidation therapy. Surgery is also a key component of induction, typically performed after several cycles of chemotherapy (19). The goal is a complete resection of the primary tumor, however, a 90% resection is acceptable in order to minimize surgical complications such as major vascular injury or end-organ damage (20). Despite aggressive induction therapy, approximately 10% of patients experience disease progression and only 20% achieve a complete response. Improving induction therapy outcomes are a major focus of ongoing clinical trials in HRNB (21).

#### 2.2.2 Consolidation phase

In the consolidation phase, the primary aim is to eradicate any residual disease. Patients undergo autologous stem cell transplantation (ASCT) and radiation therapy. During ASCT, patients receive high doses of myelosuppressive chemotherapy, followed by stem cell reinfusion to restore bone marrow function. Studies show improved outcomes for patients who undergo ASCT compared to those who receive continued chemotherapy (22, 23). After ASCT, 21.6 Gy of external beam radiation is administered to the primary tumor and any sites of persistent disease (24). While neuroblastoma is radiosensitive, there is ongoing research to reduce radiation exposure due to its associated long-term toxicity.

#### 2.2.3 Post-consolidation phase

The post-consolidation phase focuses on preventing relapse and includes multimodal therapies such as immunotherapy and isotretinoin, which will be discussed in detail in later sections. However, despite the intensive multimodal approach, outcomes for relapsed and refractory neuroblastoma remain poor. Surgery, radiotherapy, and chemotherapy, while effective in some cases, carry significant side effects and chemotherapy is limited by the development of treatment resistance (25). Additionally, HRNB treatments are associated with long-term toxicities that can severely affect patients’ quality of life, leading to increased morbidity and mortality. Nevertheless, development of targeted therapies, such as anti-GD2 immunotherapy’s success, highlights the potential of such an approach to not only improve survival rates but also reduce toxic side effects, offering a better quality of life for HRNB patients (25).

### 2.3 Risk stratification and key molecular factors in HRNB

The molecular biology of neuroblastoma plays a crucial role in both clinical risk stratification and the development of targeted therapies (26). Neuroblastoma is characterized by somatically acquired genetic alterations that lead to changes in gene expression and correlate with tumor aggressiveness. Key genetic drivers of neuroblastoma include MYCN amplification, anaplastic lymphoma kinase (ALK) mutations or amplifications, telomerase reverse transcriptase (TERT) rearrangements, alpha-thalassemia/mental retardation X-linked (ATRX) deletions or mutations, and segmental chromosomal aberrations (SCAs).

MYCN amplification, the predominant genetic alteration associated with HRNB, occurs in approximately 20% cases and marks aggressive disease (27). MYCN amplification is linked to tumor metastasis, poor prognosis, and treatment challenges (28, 29). Other critical genetic changes include 1p loss of heterozygosity, commonly associated with unresectable metastatic disease and MYCN amplification, and 11q deletion, which correlates with advanced disease stages and poor survival (30–32). ALK mutations are also frequently found in HRNB, and ALK amplification is independently associated with aggressive tumor features such as tumor progression and therapy resistance (33).

ATRX is a frequently mutated tumor-suppressor gene, including in neuroblastoma, where it plays a critical role in chromatin remodeling by regulating chromatin state, gene expression, and DNA damage repair (34). ATRX mutations are commonly found in high-risk neuroblastoma patients and are associated with a poor prognosis (35). Genomic aberrations such as recurrent ATRX deletions and inactivation lead to alternative lengthening of telomeres (ALT), a mechanism that cancer cells use to bypass telomere shortening and maintain telomere length. Another critical mechanism for telomere maintenance is the activation of telomerase via TERT expression. TERT plays a major role in maintaining chromosomal stability by regulating telomere length, thus enabling cancer cells to evade senescence and continue proliferating (36). In HRNB, whole-genome sequencing revealed structural rearrangements of TERT in 17 of 75 cases, which were linked to increased TERT expression and associated with a very poor prognosis (37).

SCAs have emerged as an important risk factor in neuroblastoma. These structural genetic alterations are associated with aggressive tumor behavior, poor prognosis, and a higher likelihood of relapse independent of the poor prognosis associated with MYCN amplification. As part of the revised neuroblastoma risk classification system, SCAs help refine prognostic stratification, identifying patients who may require more intensive therapeutic strategies (14). Additionally, epigenetic modifications in high-risk neuroblastoma, such as DNA methylation, histone modification, and non-coding RNAs, significantly contribute to tumor progression. MicroRNAs (miRs), which regulate gene expression by targeting mRNA transcripts, are often dysregulated in HRNB and their roles in tumorigenesis and as biomarkers are an area of active research (38, 39).

### 2.4 Signaling pathways in HRNB

**Figure 2:**
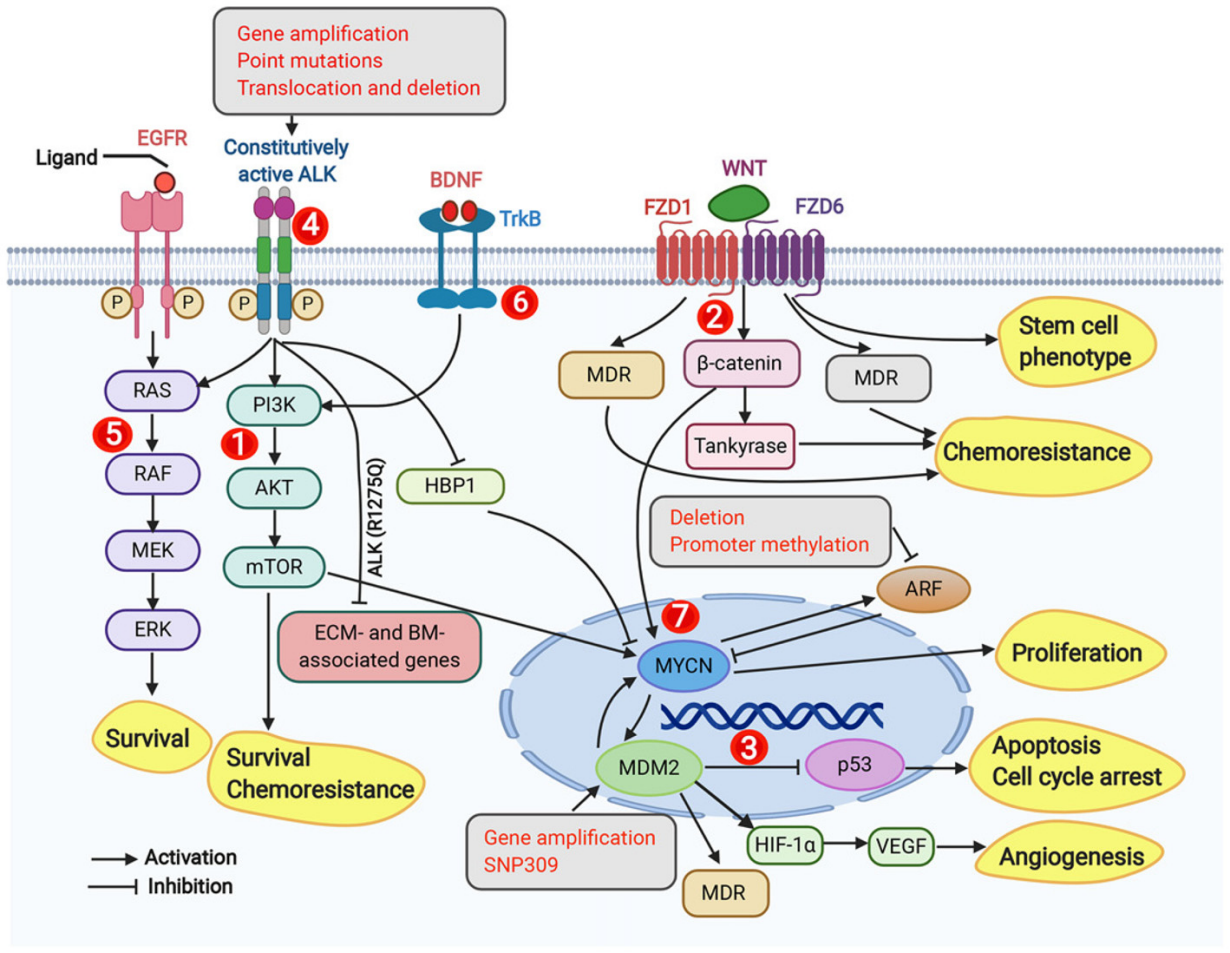
Dysregulated signaling pathways, genomic, and molecular targets in neuroblastoma. This figure from Zafar et al. (2020) highlights key molecular signaling pathways, genomic targets, and molecular targets implicated in neuroblastoma, emphasizing the complex network of dysregulated pathways, including PI3K/AKT/mTOR, Wnt, p53-MDM2, ALK, RAS-MAPK, TrkB, and MYCN, that drive tumor survival, chemoresistance, and progression. These targets represent potential therapeutic interventions for neuroblastoma treatment. Adapted from Zafar et al., 2021, Medicinal Research Reviews, 41(2):961–1021 with permission from Wiley Periodicals LLC.

Several signaling pathways play significant roles in HRNB. The most common of these pathways are the ALK and Ras-Mitogen activated protein kinase (RAS-MAPK) pathways (40, 41). Mutations in ALK, found in 12-14% of HRNB cases, activate downstream pathways PI3K/Akt and MAPK, promoting tumor cell survival and proliferation (42, 43). PI3K/Akt and Wnt/β-catenin pathways are crucial for tumor growth, while the Wnt pathway is involved in the maintenance and proliferation of neural crest cells (44, 45). Mutations in key signaling pathways, such as PI3K/Akt and Wnt/β-catenin, are common in relapsed neuroblastomas, suggesting their clinical significance (44, 45). Sonic Hedgehog (SHH) pathway is notable for maintaining neuroblastoma tumorigenicity and promoting cell proliferation (46). NF-kB activation contributes to tumor growth, metastasis, and chemokine receptor (CXCR4) upregulation, enhancing invasion (47, 48). TGF-β and Notch pathway is also important where TGF-β suppresses cell growth while Notch signaling inhibits tumor cell proliferation (49, 50). Finally, dysregulation of the p53-MDM2 pathway contributes as MDM2 overexpression leads to the degradation of p53, inhibiting tumor suppression and increasing MYCN activity, thus contributing to poor prognosis and resistance to therapy (51, 52). These collectively disrupted pathways highlight the complexity of HRNB pathobiology and suggest potential targets for novel therapies (**Figure 2**).

## 3 Current research on targeted therapies for HRNB

### 3.1 Immunotherapy

A major challenge in treating neuroblastoma is its ability to evade the immune system. This resistance is due to neuroblastoma’s low immunogenicity profile, resulting in fewer neoantigens for immune cells to target (53). Furthermore, neuroblastoma’s tumor microenvironment (TME) is characterized by low levels of tumor-infiltrating lymphocytes (TILs) and the presence of immune-suppressing cells (54, 55). These factors hinder immune responses, and research efforts to enhance HRNB’s immunogenicity and improve immune-based therapies are ongoing.

#### 3.1.1 GD2 antigen

GD2 is a ganglioside found on the outer cell membrane of peripheral neurons and the central nervous system, and skin melanocytes (25). It is highly expressed on the surface of neuroblastoma cells compared to normal cells, making it an attractive target for HRNB immunotherapy (25). GD2-targeting monoclonal antibodies (mAbs), such as Dinutuximab (ch14.18; Unituxin), were the first immunotherapy approved for use in HRNB. Dinutuximab a murine-chimeric mAb, binds to GD2, triggering the binding of C1q and initiating complement-dependent cytotoxicity (CDC) (56). This process engages the body’s immune system, facilitating antibody-dependent cell-mediated cytotoxicity (ADCC) by NK cells and granulocytes, leading to tumor cell destruction. Additionally, the downregulation of the PI3K/Akt/mTOR signaling network has been observed upon the binding of mAbs to GD2 (57). Combining Dinutuximab with IL-2, granulocyte-macrophage colony-stimulating factor (GM-CSF), and isotretinoin was a major breakthrough in HRNB treatment, increasing event-free survival (66 ± 5% vs. 46 ± 5%) and overall survival (86 ± 4% vs. 75 ± 5%) at two years (57). However, Dinutuximab therapy comes with significant side effects, primarily capillary leak syndrome, hypersensitivity reactions, and severe pain, due to GD2 expression on normal nerve cells. An alternative, Dinutuximab beta (ch14.18/CHO), produced by re-cloning in Chinese hamster ovary (CHO) cells, exhibits a more favorable glycosylation pattern, reducing immune responses and improving tolerability (58). While similar to ch14.18 in efficacy, Dinutuximab beta has been associated with reduced pain and was approved by the European medicines agency in 2017 for HRNB treatment (55). However, pain reduction was insufficient for escalating doses or exploring alternative administration methods (59).

Murine antibodies, including Dinutuximab, can also induce severe infusion reactions due to immunogenicity, specifically human anti-mouse antibodies (HAMA). To address this, humanized monoclonal antibodies targeting GD2 have been developed. Naxitamab (Hu3F8-IgG1), a humanized version of the monoclonal antibody murine 3F8 that targets GD2, was granted FDA approval in 2020 for treating relapsed or refractory HRNB in children aged one and older and adults with bone or bone marrow involvement (60). Naxitamab demonstrated cytolytic activity against neuroblastoma cells *in vitro* and led to objective responses in 34% to 45% of patients with refractory advanced neuroblastoma (60). Another humanized antibody, hu14.18K322A (hu14), modified from another murine monoclonal antibody (14G2a) that targets GD2, shows reduced complement-dependent cytotoxicity while retaining ADCC activity and synergizes with common chemotherapies *in vitro* (61, 62). In a Phase II study, combining hu14 with chemotherapy, IL-2, and GM-CSF resulted in improved early objective responses (66.7% vs. 39.1%) and a 73.7% 3-year event-free survival rate, while similar adverse effects were observed (62).

Due to the severe adverse effects of GD2 monoclonal antibodies, researchers are exploring other methods to target GD2, enhance its expression on HRNB cells, or increase antibody sensitivity. A study by Galassi et al. (63), found that increasing GD2 expression using Nanofenretinide, a nanoformulation of fenretinide, alongside Naxitamab, enhanced cytotoxicity *in vitro*. However, increasing GD2 expression alone did not always improve efficacy, as shown when combining Nanospermidine with Naxitamab, which did not increase cytotoxicity despite elevated GD2 levels. Another promising approach is radioimmunotherapy, where GD2-targeting antibodies deliver radioactivity to HRNB tumor sites (64). One recent example is the use of radio-labeled Dinutuximab, that can bind to GD2 and offer high tumor-to-organ dose ratios. However, it is important to consider that prolonged blood residence leads to absorption in blood-rich organs such as the liver, spleen, and kidneys (64).

**Figure 3:**
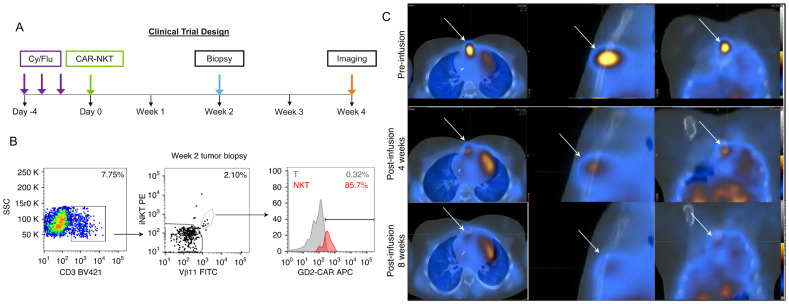
CAR-NKT cells infiltrate tumor sites and mediate tumor regression in refractory neuroblastoma patients. **Figures 2** and Extended Data **Figure 1** from Heczey et al. demonstrate that NKT cells are present at higher frequencies within tumor-infiltrating lymphocytes compared to peripheral blood lymphocytes, with 85.7% of tumor-infiltrating NKT cells expressing CAR-GD2 **(B)**. Panel **(C)** illustrates the reduction in size of neuroblastoma bone metastasis. Panel **(A)** depicts the clinical trial design for this study. These findings highlight the therapeutic potential of CAR-NKT cells in treating neuroblastoma tumors and promoting their regression. Adapted from Heczey, A., Courtney, A.N., Montalbano, A., et al. Anti-GD2 CAR-NKT cells in patients with relapsed or refractory neuroblastoma: an interim analysis. Nat Med 26, 1686–1690 (2020).

Furthermore, GD2’s high expression on neuroblastoma cell surfaces has made it a target for chimeric antigen receptor T-cells (CAR-T). CAR-T cell therapy directs engineered T cells to a specific antigen, such as GD2, via chimeric antigen receptors (65). Although CAR-T therapy targeting GD2 in neuroblastoma shows promise, it faces limitations such as T cell exhaustion, loss of functionality, and poor tumor infiltration. A Phase I trial (NCT02107963) demonstrated the feasibility and safety of GD2-CAR T-cell therapy, with no dose-dependent cytotoxicity (66). While GD2-CAR T-cells expanded in all patients, their persistence was limited, and all patients eventually progressed (67). Research suggests that expansion is influenced by factors like CXCR3+/– expression, with better expansion associated with naive T cells and CXCR3+ monocytes (68). In response to the challenges faced by CAR-T therapy arising from the intrinsic limitations of T-cells, CAR-NK T cells are being explored as an alternative to CAR-T cells alone. This leverages the unique antitumor activity of Natural Killer-T cells. These cells have been shown to expand and localize to tumors in relapsed neuroblastoma patients (**Figure 3**) (69). In a Phase I trial (NCT03294954), three patients underwent lymphodepleting conditioning followed by CAR-NK T cells infusion, with no dose-limiting toxicities (69, 70). One patient exhibited an objective response with regression of bone metastatic lesions. These promising results suggest that CAR-NKT cells can be expanded to clinical scale and safely used for HRNB treatment and beyond (70).

GD2 remains a critical therapeutic target for HRNB, with multiple strategies, including monoclonal antibodies, radioimmunotherapy, and CAR therapies, and the introduction of anti-GD2 immunotherapy notably improved survival for HRNB. However, the toxicities associated with currently approved immunotherapies are significant and limit the current clinical treatment strategies so further research to optimize GD2-targeted therapies for HRNB.

#### 3.1.2 Glypican-2

**Figure 4:**
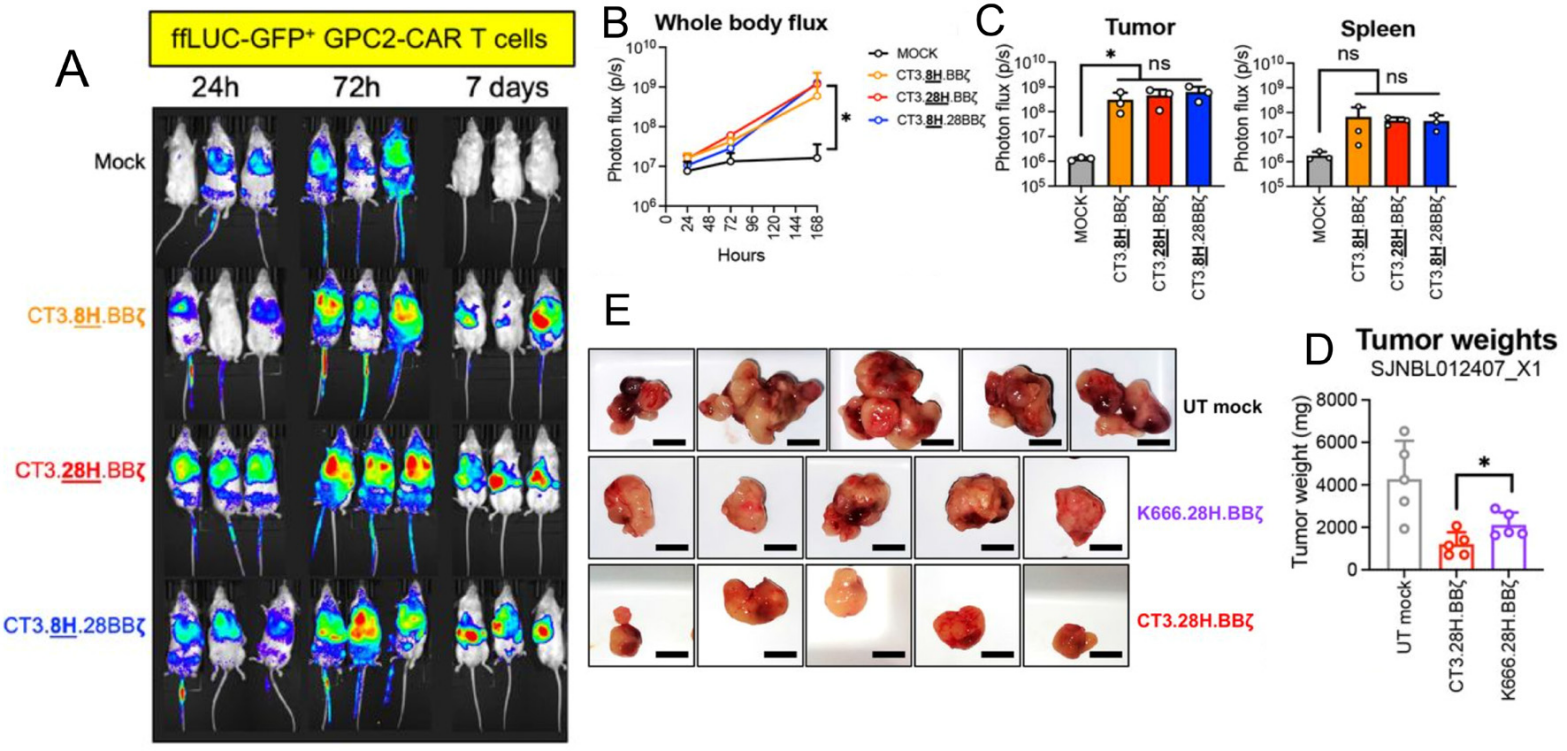
Preclinical evaluation of GPC2-targeting CAR T cells for neuroblastoma treatment. **(A-C)** show that GPC2-CAR T cells efficiently home to the tumor microenvironment (TME), expand, and enrich as a cytotoxic effector population, highlighting their potential for targeted tumor eradication. **(D, E)** compare the performance of GPC2-CAR T cells with GD2-CAR T cells (K666.28H.BBζ), showing superior *in vitro* cytotoxicity, tumor rechallenge response, and reduced tumor burden *in vivo*. Adapted from Sun et al., 2023, Journal for ImmunoTherapy of Cancer, 11(1):e005881 with permission from BMJ Specialist Journals.

Glypican-2 (GPC2) is a heparan sulfate proteoglycan oncoprotein belonging to the glypican family that is anchored to the cell membrane via a glycosylphosphatidylinositol (GPI) anchor (71). GPC2 is expressed on the surface of neuroblastoma cells, activating the WNT signaling pathway and stimulating growth. This is correlated with worse outcomes with HRNB patients with higher GPC2 expression (71). Recent studies have highlighted differences in GPC2 transcript expression between neuroblastoma cells and normal tissues, suggesting the existence of a tumor-selective GPC2 sequence that can be targeted specifically to the tumor (72). GPC2’s selective expression to neuroblastoma makes it an attractive target for immunotherapy, particularly for CAR-T therapies, as a means to enhance neuroblastoma cell specificity and limit cytotoxicity to normal cells. Repeated testing of CAR constructs has shown that a CAR composed of a single chain variable fragment (scFv) derived from an antibody that targets GPC2 (CT3) exhibits superior anti-tumor activity against HRNB (**Figure 4**) (73). Additionally, combining GPC2 targeted CAR-T cell therapy with immunomodulatory agents like long-acting interleukin-7 (rhIL-7-hyFc) has demonstrated enhanced T-cell expansion, reduced exhaustion markers, and improved efficacy against solid tumors with low antigen density (74). While GPC2 therapy is promising there is significant heterogeneity in GPC2 expression in neuroblastoma, meaning the therapy is only effective in patients who express GPC2 (72–74). For sustained anti-tumor activity, the persistent presence of CAR-T cells is crucial. However, CAR-T cells can have a limited lifespan *in vivo*, which can impact their long-term effectiveness (72–74).

#### 3.1.3 B7-H3 (CD276)

B7-H3 is a checkpoint inhibitor ligand that is overexpressed in neuroblastoma cells. Studies have shown that B7-H3 contributes to tumorigenesis, angiogenesis, invasion, and metastasis through various mechanisms (75, 76). Blocking B7-H3 with B7-H3 mAb (5B14) *in vitro* has enhanced the ability of NK cells to kill neuroblastoma cells (77). Recently, pre-clinical studies are exploring CAR targeting B7-H3 as a way to deliver immune cells directly to the tumor. Engineered CAR-NK92 cells targeting B7-H3 have successfully eliminated neuroblastoma cell lines in monolayer cultures and 3D models *in vitro* (78). However, genetic modification of primary NK cells are challenging due to low transfection efficiency and poor CAR function (78). Similarly, engineered CAR-T cells targeting B7-H3 show strong activity against neuroblastoma cells *in vivo* (79). For instance, SynNotch GD2-B7-H3 CAR T-cells exhibited high stability, high specificity, and enhanced cytotoxicity against neuroblastoma cells *in vitro* and in xenograft mouse models compared to conventional CAR-T cells (80). The SynNotch gating strategy, which uses GD2 as the gate and B7-H3 as the inducible target, is being leveraged to improve the precision of CAR T-cell therapies and enhancing the safety profile of using GD2 therapy alone (80). However, GD2-B7-H3 CAR-T cells showed lower cytotoxic efficiency with longer times required for gated CAR to show similar levels of cytotoxicity compared to conventional CAR-T cells (80). This can be resolved with more rounds of therapy; however other issues might arise like tumor escape due to loss of either needed antigen (80).

#### 3.1.4 NECTIN2-TIGIT axis

The Nectin cell adhesion molecule-2 and T-cell immunoreceptor with Ig and ITIM domains (NECTIN2-TIGIT) axis is an immune checkpoint involved in immune evasion. NECTIN2, a cell adhesion molecule expressed on the surface of HRNB cells, acts as a binding partner for TIGIT, an immune checkpoint receptor found on immune cells such as T cells and NK cells (81). This axis is strongly associated with the dysfunction of both NK cells and T cells. Research has shown that blocking TIGIT and programmed death-ligand 1 (PD-L1) can induce a complete response *in vivo*, including in chemotherapy-resistant neuroblastoma cells (81). Another pre-clinical study showed that blocking both PD-L1 and TIGIT increased the efficacy of Dinutuximab beta (GD2 antibody) in HRNB tumors *in vivo* causing complete removal of tumor compared to blocking either TIGIT and PD-L1 separately (82). These findings highlight the potential of targeting the NECTIN2-TIGIT axis to improve the TME and enhance the immune response in HRNB, but further research is needed to explore immunotherapy efficacy while managing the risk of immunotoxicity, prior to implementing this strategy in clinical practice (81).

#### 3.1.5 DLK 1

Delta-like homologue 1 (DLK1) is a surface protein that belongs to the epidermal growth factor (EGF)-like protein family and is believed to suppress the Notch signaling pathway (83). Recent research has identified DLK1 as a promising target for immunotherapy, particularly in neuroblastoma. DLK1 is overexpressed in neuroblastoma cells, and its expression is largely restricted to the adrenal medulla and pituitary gland (84). According to Hamilton et al. DLK1 may play a role in maintaining the undifferentiated state of neuroblastoma cells, preventing their maturation (84). Further studies have shown that depletion of DLK1 in neuroblastoma cells promotes cell differentiation. Additionally, ADCT-701, an antibody-drug conjugate currently in an ongoing phase I clinical trial (NCT06041516) for adult patients with neuroendocrine tumors, has demonstrated effectiveness in targeting and killing DLK1-positive neuroblastoma cells *in vivo* (84, 85). Further research needs to done be in order to detect off target toxicity since ADCT-701 does not bind to mouse DLK-1 (84).

#### 3.1.6 Immune checkpoint inhibitors

Immune checkpoints, such as programmed cell death protein 1 (PD-1) and cytotoxic T lymphocyte-associated protein 4 (CTLA-4), are critical regulators of the immune system, controlling its ability to recognize and eliminate tumor cells (86). Under normal conditions, these checkpoints prevent excessive immune activation, maintaining self-tolerance and preventing autoimmunity. However, many tumors exploit these regulatory mechanisms to evade immune detection, leading to immune suppression (87). One such immune checkpoint, PD-L1, is a ligand expressed on the surface of neuroblastoma cells, where it binds to PD-1 receptors on activated T cells, inhibiting T cell activation and proliferation (88). PD-L1 inhibitors such as nivolumab are currently being investigated in early-phase clinical trials. These agents work by blocking the inhibitory signals from PD-1 engagement, thus enhancing T cell activity against tumors. Early-phase clinical trials are exploring the combination of nivolumab with dinutuximab (GD2 antibody), and preliminary results suggest this combination may enhance immune-mediated tumor responses and improve overall survival in patients with relapsed or refractory neuroblastoma (89). While PD-1 signaling blockade can improve antitumor responses and lead to long-lasting clinical benefits, 30%–60% of patients do not respond to PD-1/PD-L1 inhibitors and many formed resistance to them (88).

Another checkpoint inhibitor, Cytotoxic T-Lymphocyte Antigen-4 (CTLA-4), acts as an inhibitory receptor on T cells by competing with CD28 for binding to B7-1 and B7-2 molecules on antigen-presenting cells (90). This competition negatively regulates T cell activation, contributing to immune evasion by tumors. A recent study *in vivo* demonstrated that combining CpG-PBNP-PTT (a form of photothermal therapy) with anti-CTLA-4 antibodies resulted in complete tumor regression in treated tumors and slower progression in untreated ones. This combination also led to long-term survival and the generation of protective immunity against tumor rechallenge (91). However, HRNB heterogeneity and poor immunogenicity limit the efficacy of CTLA-4 therapy (86).

### 3.2 Non-coding RNAs

#### 3.2.1 MicroRNAs

MicroRNAs (miRs) are small, non-coding RNA molecules, typically 19-23 nucleotides in length, that regulate gene expression by binding to target miRs, leading to their degradation or translational inhibition (92). Dysregulation of miRs has been implicated in neuroblastoma tumorigenesis, with known roles in cell differentiation, tumor promotion, and tumor inhibition (93). Studies have shown that manipulating miR levels—either by increasing levels with miR mimics or decreasing levels with miR inhibitors—holds therapeutic potential for treating cancers like neuroblastoma (93).

**Figure 5:**
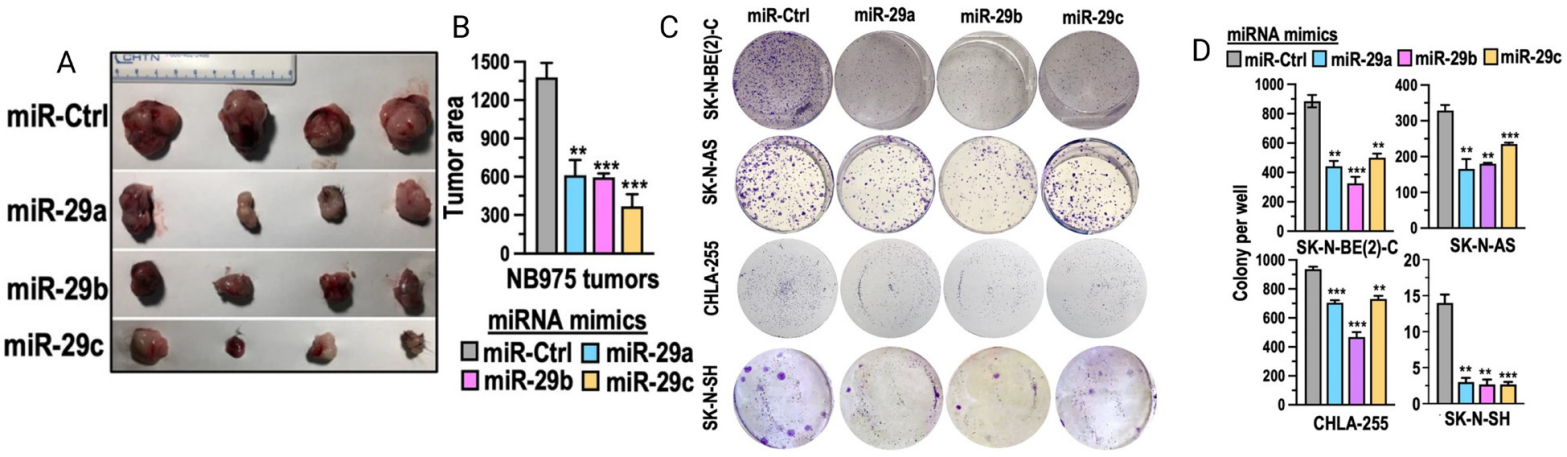
miRNA-29 demonstrates anti-tumor activity by inhibiting cell growth, colony formation, and reducing tumor size and area. **(A, B)** show tumor area reduction following miR-29 transfection *in vivo*. **(C)** depicts the inhibition of various neuroblastoma cell lines *in vitro* after treatment with miR-29. Adapted from Pathania et al., 2024, Cell Death & Disease, 15 (6):428 with permission from Springer Nature.

Recently it was found that higher levels of the miR-29 family (miR-29a, miR-29b, and miR-29c) were associated with better survival in neuroblastoma patients (**Figure 5**) (94). The mechanism of action of miR-29 is believed to involve the activation of NK cells through targeting B7-H3 in neuroblastoma (94). It was shown that neuroblastoma cells exhibited higher B7-H3 expression and lower levels of miR-29, when neuroblastoma cells were transfected with miR-29 mimics for 48 hours it resulted in a significant depletion of B7-H3 mRNA resulting in enhanced NK cell activation and cytotoxicity (94, 95). Furthermore, when activated immune cells were combined with miR-29-expressing neuroblastoma cells, there was a marked increase in CD107 and perforin expression, indicating NK cell activation, enhanced tumor lysis and higher IL-2 secretion (94, 95).

The miR-124 family is another miR group with potential implications for neuroblastoma treatment. miR-124, a conserved miR predominantly expressed in neurons, plays a key role in regulating the development and function of the nervous system (96). However, its role in neuroblastoma remains controversial, likely due to the heterogeneity of the disease (97). Nolan et al. showed that miR-124 directly targets key cytoskeletal genes, leading to a shift from a mesenchymal (invasive) phenotype to a more epithelial (less invasive) phenotype, which enhances neuroblastoma cells’ sensitivity to chemotherapy (98). This is consistent with findings by Sharif et al. and Zhao et al. which displayed that miR-124 can arrest the cell cycle at the G1 phase, preventing cell proliferation in HRNB cell lines (99, 100). In contrast, Zhang et al. found that miR-124 directly downregulates VAMP3, which was shown to promote cell death, which correlates with poorer patient outcomes in neuroblastoma (97). This is due to the interplay between miR-124 and VAMP3, which influences the expression of genes involved in various cellular processes. These findings align with Huang et al. who showed that inhibition of miR-124 led to increased AHR expression and resulted in cell differentiation in HRNB cell lines (101).

The miR-34 family is another widely studied miR family with important implications targeting MYCN. Overexpression of miR-34a has been shown to significantly reduce proliferation, induce cell cycle arrest, and activate apoptosis and levels are lower in HRNB (102). Possible targets include key oncogenes MYCN, ALK and LIN28B along with HNF4α and the autophagy-related gene 5 (ATG5) (102–104). These miRs can also enhance tumor sensitivity to chemotherapy by targeting multiple signaling pathways (103).

The let-7 miR family plays a crucial role in suppressing neuroblastoma tumors by targeting several oncogenes (DICER1, ARID3B, MYC, HMGA2) (105, 106). However, let-7 is frequently downregulated in neuroblastoma through multiple mechanisms. One mechanism involves the LIN28B protein, which is often overexpressed in high-risk neuroblastoma (HRNB) and associated with poor patient outcomes (107). LIN28B suppresses let-7 by increasing MYCN protein levels, which in turn targets and inhibits Let-7 (107). This LIN28B-MYCN pathway is associated with disrupting neuroblast differentiation (107). Furthermore, MYCN has been shown to suppress let-7 independently of LIN28B. In high-risk neuroblastoma (HRNB) cells with LIN28B knocked down, where MYCN levels were elevated, let-7 miRNA expression was still suppressed (106). This is because excess MYCN mRNA can directly bind to and degrade let-7 (106). Intriguingly, the frequency of let-7 gene loss differs between MYCN-amplified and non-amplified tumors (106). Loss of let-7 genes is common in non-MYCN-amplified tumors (63.4%), but much less frequent in MYCN-amplified tumors (16.7%) (106). This suggests that in MYCN-amplified tumors, the excess MYCN protein functionally sequesters and inactivates let-7, thus reducing the selective pressure for the tumor cells to physically delete the let-7 genes (106).

While miR-29, miR-124, miR-34, and miR Let-7 are commonly recognized as neuroblastoma-related miRs, there are many other miRs currently under investigation and our understanding of the role of miRs in cell proliferation and differentiation in neuroblastoma is rapidly evolving. miRs have demonstrated successful reduction of tumor cell proliferation in pre-clinical studies of neuroblastoma and other cancers, but clinical studies have been limited by cytotoxicity.

Furthermore, miRs are actively being explored as potential biomarkers for disease surveillance and prognostic indicators. Further research is needed to explore how miRs can be combined with other therapeutic strategies for improved treatment outcomes.

#### 3.2.2 Long non-coding RNA

Long noncoding RNAs (lncRNAs) are a class of RNA molecules that range in length from 200 nucleotides to 100 kilobases and do not encode proteins (108). Despite this, they play crucial roles in transcriptional and epigenetic regulation of gene expression (109). LncRNAs have been implicated in various cancers, where they regulate tumor-associated genes and other cellular processes (110). For example, the metastasis-associated lung adenocarcinoma transcript (MALAT1) is upregulated in neuroblastoma cells under hypoxic conditions compared to normal conditions (111). MALAT1 promotes the upregulation of Fibroblast Growth Factor 2 (FGF2) mRNA and protein expression in neuroblastoma cells and enhances FGF2 secretion into the extracellular fluid. This leads to endothelial cell migration, invasion, and vasculature formation. Additionally, MALAT1 has been shown to have a significant positive relationship with Axl, a transmembrane receptor tyrosine kinase (RTK) that acts as an oncogene and is overexpressed in neuroblastoma (112). Bi et al. demonstrated that targeting Axl with its inhibitor has been shown to suppress neuroblastoma cell metastasis by regulating tumor-driven angiogenesis, which highlights its potential as a promising therapeutic target for neuroblastoma treatment (112).

Small nucleolar RNA host gene-7 (SNHG7) is a lncRNA that is upregulated in neuroblastoma, where it plays a significant role in cell proliferation and is associated with poor prognosis. SNHG7 is suggested to promote cell proliferation, migration, and invasion by modulating miR-653-5p, a miR that inhibits STAT2 (113). STAT2 is involved in promoting the expression of oncogenic gene STAT3 (114). Thus lncRNA SNHG7 induces STAT2 overexpression which contributes to neuroblastoma progression (113). *In vivo* studies show that silencing SNHG7 significantly inhibits tumor growth in xenograft models, leading to an increase in miR-323a-5p and miR-342-5p expression (115). Additionally, silencing SNHG7 was shown to enhance Cisplatin sensitivity (116). SNHG7 acts as a sponge for miR-329-3p, which plays a role in reducing Cisplatin resistance and autophagy inhibition, resulting in the upregulation of MYO10 which plays a role in reducing Cisplatin sensitivity (116). Further research is essential to better understand how lncRNAs interact with other molecular targets and how they can be integrated with complementary therapeutic strategies to enhance treatment outcomes.

#### 3.2.3 Circular RNA

Circular RNAs (circRNAs) are mainly non-coding RNAs, typically 19–23 nucleotides in length, that play important roles in cell regulation (117). Their name is derived from the unique structure they form by binding the ends of the RNA together to create a circular polynucleotide, which enhances their stability and makes them resistant to exonucleases (117). Pre-clinical *in vivo* studies have shown that circRNAs can influence both tumor survival and suppression in HRNB, depending on their target (118–122). **Table 1** provides examples of different circRNAs and their roles in HRNB. Further research is needed to explore their potential as a promising therapeutic target for neuroblastoma treatment.

**Table 1:** Examples of circular RNAs and their effects in HRNB.

Examples of Circular RNA, Targets, and Effects			
Circular RNA	Availability in HRNB	Target	Effects
Circ_0000285	Upregulated in HRNB	Inhibits miR-582-3p	Enhances Wnt/β-catenin signaling, promoting tumor survival (120).
Circ_0135889	Upregulated in HRNB	Inhibits miR-127-5p	Upregulates Nerve Differentiation 1 (NEUROD1), promoting tumorigenesis and enhancing proliferation (118).
Circ_0001361	Upregulated in HRNB	Inhibits miR-490-5p	Upregulates IGF2 and promotes tumor survival (122)
CircPDE5	Upregulated in HRNB	Inhibits miR-362-5p	Upregulates NOL4L and promotes tumor survival (121)
.Circ-SHPRH	Downregulated in HRNB	Induces P21, a protein part of WAF/CIP/KIP family of cyclin-dependent kinase inhibitors.	Promotes tumor suppression by inhibiting Cyclin-dependent kinases complex activation through SHPRH-146aa protein (119)

The table presents the names of circRNAs, their presence in HRNB, associated targets, and their impact on tumor survival.

### 3.3 Targeting pathways/molecular biomarkers

#### 3.3.1 ALK inhibitors

Genomic aberrations of the ALK gene are found in approximately 14% of patients with HRNB (123); therefore it is a critical potential target for therapeutic development. Crizotinib, the first ALK inhibitor to undergo clinical assessment, was evaluated in two phase III trials which confirmed its efficacy over standard first-line chemotherapy, leading to its FDA approval in 2011 (124–126). In 2013, a phase I trial was initiated in which crizotinib was administered with chemotherapy (127) and subsequently expanded to phase III trial due to promising results (128).

However, early clinical trials of first-generation ALK inhibitors, like crizotinib, demonstrated limited efficacy (129). More potent second- and third- generation ALK inhibitors, such as ceritinib, lorlatinib, brigatinib, alectinib, and repotrectinib, have shown improved therapeutic activity in neuroblastomas with ALK mutations, though they achieve complete responses in only a small number of patients (130–135).

Among these, lorlatinib, a third-generation ALK inhibitor, was designed to overcome resistance and shows superior potency compared to crizotinib, particularly against three of the most common ALK mutations (132, 136–138). Despite these promising results, treatment resistance remains a significant clinical challenge with prolonged use of these targeted therapies.

#### 3.3.2 FAK inhibitors

In addition to ALK inhibitors, focal adhesion kinase (FAK) inhibitors are of interest. A preclinical study using patient-derived xenografts of neuroblastoma evaluated the effects of FAK inhibitors PF-573, 228 and Y15. The inhibitors significantly reduced cell survival, proliferation, and motility, while inducing cell cycle arrest. FAK inhibition also decreased tumor sphere formation and reduced the expression of stem cell markers, suggesting a reduction in cancer stem cell-like properties. These results position FAK as a promising therapeutic target for neuroblastoma (139).

Chugh et al. evaluated the dual ALK and FAK inhibitor ESK440 in preclinical HRNB models (140). ESK440 significantly inhibited cell proliferation *in vitro* with ALK aberrations, and reduced ALK, FAK, and downstream target activation, impairing migration and invasion. ESK440 significantly inhibited cell proliferation *in vitro* with ALK aberrations, and reduced ALK, FAK, and downstream target activation, impairing migration and invasion (140). Importantly, ESK440 also reduced MYCN levels. In neuroblastoma cell lines and patient-derived xenografts, ESK440 treatment reduced tumor growth with no observed toxicity. ESK440 showed comparable or enhanced efficacy over lorlatinib and maintained effectiveness in lorlatinib-resistant models (140). These findings suggest that ESK440 is a promising targeted therapy for ALK-driven neuroblastoma and warrant further clinical investigation.

#### 3.3.3 MYCN inhibitors

MYCN is the hallmark oncogene of neuroblastoma and is a prognostic factor that is indicative of unfavorable outcomes in patients with HRNB. MYCN has been described as a transcription factor that controls several cellular processes. Many strategies have been proposed to directly target MYCN; however, translation to clinical therapeutics have not yet been successful.

An approach to target MYCN is through the use of ornithine decarboxylase 1 (ODC1) inhibitors because the ODC1 gene encodes the rate-limiting enzyme ODC involved in the biosynthesis of polyamines (141). Polyamines are well-studied cationic molecules integral to normal and cancer cell growth and their biosynthesis pathway is a well-characterized direct Myc transcription target (142). A study by Rounbehler et al. demonstrated the upregulated polyamine biosynthesis in MYCN-amplified neuroblastoma contributes to aggressive tumor growth (143). The study further demonstrated the significance of the MYCN and polyamine pathway in neuroblastoma tumorigenesis by using difluoromethylornithine, an ODC inhibitor, which decreased polyamine levels. The decrease of polyamine levels disabled Myc’s ability to drive cell growth and slow the cell cycle.

Inhibition of Aurora A (AURKA) and Aurora B kinases (AURKB) has been widely researched and is currently a potential way to target MYCN. The family of aurora kinases are associated with centrosome separation in the cell cycle (144). AURKA complexes with MYCN during the S-phase of cell division leading to MYCN stabilization and decreased susceptibility to proteasomal degradation (145). The same study by Roeschart et al. showed that combinatorial inhibition of Aurora-A and ataxia telangiectasia and Rad3 related (ATR) kinase which suppresses double-strand break accumulation, results in regression of MYCN-amplified tumors. AURKB is a direct transcriptional target of MYCN and both aurora kinases have been correlated with a poor prognosis in neuroblastoma (146, 147). Neuroblastoma cells have been treated with alisertib, a specific AURKA inhibitor, and were shown to inhibit cell growth, G2/M arrest, degradation of MYC and tumor growth inhibition (148).

#### 3.3.4 Cyclin-dependent kinase inhibitors

One of the hallmarks of cancer is a dysregulation in cell division which leads to aberrant cell proliferation; inhibiting cell division is a common approach for cancer therapy. Cell division is controlled by a complex of cyclin and cyclin-dependent kinases (CDKs) (149), and in neuroblastoma, CDK4/6 inhibitors are of particular interest. Ferguson et al. demonstrated that CDK4/6 inhibitor palbociclib was able to inhibit proliferation and promote extensive neuronal differentiation of adrenergic neuroblastoma cells *in vivo* (150). The first clinical study exploring a CDK4/6 inhibitor in a pediatric population utilized ribociclib, an orally bioavailable and highly specific CDK4/6 inhibitor (151). The study demonstrated that ribociclib was well-tolerated in pediatric patients, but its efficacy in HRNB specifically remains unclear due to the complex molecular heterogeneity of neuroblastoma tumors. Further studies must be conducted to determine the specific efficacy, optimal patient population, and long-term benefits of ribociclib in HRNB patients.

#### 3.3.5 PHOX2B inhibitors

The PHOX2B (paired like homeobox 2B) gene is essential in the development of the autonomic nervous system, promoting neural crest cell differentiation, and PHOX2B mutations are associated with peripheral neuroblastic tumors such as neuroblastoma (4, 152). In fact, it was the first gene for which germline mutations were discovered in neuroblastoma (153) and low expression of PHOX2B is associated with a higher tumor stage, poor outcome and poor survival (154). PHOX2B binds to the ALK gene promoter (155) and activates the Delta-Notch pathway to induce neuroblastoma cell proliferation (156, 157). Mycophenolate mofetil and XAV939 are two molecules that have demonstrated potential to inhibit PHOX2B in neuroblastoma cells in preclinical studies. Di Zanni et al. showed that mycophenolate mofetil decreases PHOX2B mRNA and protein expression in neuroblastoma cells *in vitro* (157), while Suebsoonthron et al. shows PHOX2B downregulation with XAV939 treatment. Additionally, XAV939 increases chemosensitivity of neuroblastoma cell lines to doxorubicin, a common chemotherapeutic associated with significant treatment toxicity (158).

Some limitations that must be addressed before advancing the targeting of PHOX2B in neuroblastoma treatment include the need for more selective inhibitors to avoid off-target effects (157), the potential for resistance mechanisms to develop (158), the variability in tumor responses (157), and the requirement for more comprehensive *in vivo* studies to assess long-term safety and efficacy (158). Additionally, the complex interaction between PHOX2B and other signaling pathways in neuroblastoma suggests that combination therapies may be necessary, which could introduce challenges in terms of optimizing treatment regimens and minimizing toxicity (158).

#### 3.3.6 P53-mouse double minute 2 homolog pathway Inhibitors

MDM2 inhibitors have demonstrated *in vitro* and *in vivo* anticancer activities (159, 160). Nutlins are small molecule MDM2 inhibitors that can induce cell cycle arrest or apoptosis in p53 wildtype cancer cells (161). Peirce and Findley demonstrated Nutlin-3’s ability to sensitize p53-null neuroblastoma cells to doxorubicin (162), but such first-generation MDM2 inhibitors were limited by poor bioavailability and pharmacokinetics, which hindered their *in vivo* efficacy (163).

RG7388, or idasanutlin, is a second-generation Nutlin developed to improve efficacy and toxicity of the Nutlins. Treatment with RG7388 induces functional activation of the p53 pathway and apoptosis of neuroblastoma cell lines *in vitro (* 164 *)*, and combination therapy with RG7388 treatment and chemotherapeutics demonstrated increased apoptosis in p53 wild-type neuroblastoma cells (165). An active phase I/II clinical study (NCT04029688) is investigating the safety and efficacy of RG7388 for solid tumors, including neuroblastoma (166).

#### 3.3.7 PI3K/Akt/mTOR pathway

The aberrant activation of the phosphatidylinositol 3’-kinase (PI3K)/Akt/mammalian target of rapamycin (mTOR) pathway serves as a pro-survival signaling cascade (167). Pathologic activation of Akt has been shown to frequently occur in neuroblastoma and is associated with a poor prognosis and is possibly related to MYCN amplification (168). Strong phosphorylation of the S6 ribosomal protein (a target of mTOR) was also observed in neuroblastoma samples; therefore, the PI3/Akt/mTOR pathway affects several pathways or proteins promoting a more aggressive cancer cell phenotype. Notably, the PI3/Akt/mTOR pathway contributes to MYCN oncogene stabilization which is associated with cell proliferation (169).

A study by Chesler et al. (170) has shown that the PI3/Akt/mTOR pathway inhibition leads to a decrease in the levels of N-Myc protein in NB. Furthermore, a clinical study (NCT00776867) evaluating the efficacy of perifosine (Akt inhibitor) was conducted in pediatric patients with solid tumors including HRNB (NCT00776867). In this phase I/Ib trial, 27 HRNB patients were treated with the AKT inhibitor perifosine, with nine MYCN-non-amplified patients remaining progression-free for up to 74 months, including one complete response. Toxicity was minimal even with extended treatment (11-62 months) (171). The study supports perifosine as a potential therapy for MYCN-non-amplified HRNB, either as monotherapy or in combination with other treatments. However, limitations such as the small sample size, lack of a control group, and the inclusion of only one patient with MYCN-amplified HRNB need to be overcome to improve the generalizability of the results.

#### 3.3.8 Eukaryotic initiation factors

Eukaryotic initiation factors (eIFs) are essential proteins that regulate the initiation phase of translation, the process by which ribosomes synthesize proteins from mRNA templates (172). These factors, including eIF1, eIF2, eIF3, eIF4, and eIF5, facilitate the assembly of the translation machinery and ensure accurate protein synthesis (173). As mentioned in previous sections, MYCN gene is amplified and a marker of HRNB. The 5’ untranslated region (UTR) of MYCN mRNA plays a crucial role in its translation by interacting with eIFs, thereby influencing MYCN protein levels.

Additionally, rocaglates, a class of compounds that stabilize eIF4A, have been shown to inhibit translation initiation by forming steric barriers that block initiation ribosomes (174). Amidino-rocaglates, synthetic derivatives of rocaglates, are among the most potent inhibitors in this class (175). CR-1-31-B, a small molecular rocaglate compound, functions as an inhibitor of eukaryotic translation initiation by targeting eIF4A, an RNA helicase that unwinds RNA secondary structures for translation initiation. A study by Skofler et al. is a preclinical *in vitro* investigation that explores the potential of targeting eIF4A1 as a novel therapeutic approach for neuroblastoma (176). They found that eIF4A1 is overexpressed in neuroblastoma tissues, contributing to tumorigenesis through its role in translation initiation. By using the synthetic rocaglate CR-1-31-B, which clamps eIF4A1 to mRNA and inhibits translation initiation, they observed decreased cell viability, increased apoptosis, and alterations in cell cycle distribution in neuroblastoma cell lines. Notably, CR-1-31-B showed effectiveness at low nanomolar doses, without affecting non-malignant cells, highlighting the therapeutic potential of targeting translation initiation in neuroblastoma (176). A recent preclinical *in vitro* study by Volegova et al. also demonstrated that inhibiting eIF4A1, a key initiation factor, using the compound CMLD012824, led to growth inhibition in MYCN-amplified neuroblastoma models without generalized toxicity (177).

Targeting eukaryotic initiation factors (eIFs) has shown therapeutic potential in HRNB but several limitations must be addressed before moving into clinical studies. Challenges such as off-target effects, the need for more selective inhibitors, potential toxicity, and the complexity of targeting translation initiation *in vivo* must be overcome to ensure the specificity, safety, and efficacy of eIF-targeted therapies. These factors are critical to consider for advancing eIF-targeting strategies from preclinical research to clinical application.

## 4 Summary and future directions

HRNB is a devastating pediatric cancer prone to aggressive tumors, metastatic disease, and development of treatment resistance. Despite multimodal therapy and decades of research, the overall survival remains poor, highlighting the need for more effective therapies. A more targeted approach to therapy could potentially increase treatment efficacy while simultaneously reducing treatment side effects, which can be significant and life-limiting. Current approaches for targeted therapies for neuroblastoma include targeting genetic aberrations, disrupted signaling pathways, epigenetic regulators, and Bcl-2 family proteins. While immunotherapies such as GD2 antibody-based treatments have become a standard treatment option for neuroblastoma, their optimization and alternative immunotherapy technologies such as CAR T-cell therapy are areas of ongoing research.

To bridge the gap and improve translational research, more physiologically relevant and technically reproducible model systems are needed. Most *in vivo* neuroblastoma model systems consist of mice, zebrafish, or less commonly, chick chorioallantoic membrane (CAM) (178). Each of these models may answer specific scientific questions but cannot completely reproduce the human TME, creating a significant gap in research efforts and a lack of translation from pre-clinical studies into successful clinical developments (178). Novel methods such a patient-derived xenograft models, humanized mouse models, and 3D tissue-engineered system may be more relevant models in which to test neuroblastoma therapeutics (178).

In parallel with the development of better models, personalized medicine based on patient-specific genetic profiles holds promise for enhancing treatment efficacy. Studies identifying genomic variations at the time of relapse to determine optimal combination of small molecule inhibitors treatments are underway and strongly supported by preclinical studies. Additionally, miRs represent ways to study individual tumor biology and leverage functional miRs as monotherapy, combination therapy, or to establish synergism with existing therapies such as chemotherapy, immunotherapy, and radiation therapy. Furthermore, liquid biopsies utilizing cell-free DNA can potentially detect genetic alterations not captured by a single-site biopsy, providing insights into tumor heterogeneity and expanding actionable targets for therapy (179).

Additionally, strategies to enhance delivery of therapeutics directly to the tumor are another area of active research. One approach is liposomal drug delivery, where drugs are encapsulated in liposomes decorated with molecules or ions that specifically target neuroblastoma cells (180). Other methods, such as carbon nanotubes, drug-loaded silk films, amphiphilic diblock polymers, and nanoparticles, can also be functionalized with targeting ligands to improve drug delivery efficiency (180). Another promising delivery method involves mesenchymal stem cells (MSCs), which can migrate to tumors and release growth factors to modulate the tumor microenvironment. MSC-derived extracellular vesicles are being explored as drug delivery vehicles due to their ability to efficiently load anticancer agents and target tumor cells, offering a potential avenue for targeted therapy in solid tumors (181).

Ultimately, understanding neuroblastoma’s genetic complexities can create the ability to personalize medicine based on unique tumor characteristics. As mutants arise and new signaling pathways are identified, novel therapies targeting these pathways can be explored. Research determining the mechanism of treatment resistance and strategies to overcome treatment resistance will be critical areas for neuroblastoma research to continue progress improving the dismal outcomes associated with HRNB. Striving to improve overall survival while also reducing treatment associated toxicity and side effects is the driving force behind ongoing research efforts, with the hope of significantly improving both survival rates and quality of life for children diagnosed with HRNB.
